# Baicalin alleviates chronic obstructive pulmonary disease through regulation of HSP72-mediated JNK pathway

**DOI:** 10.1186/s10020-021-00309-z

**Published:** 2021-05-30

**Authors:** Dexun Hao, Yanshuang Li, Jiang Shi, Junguang Jiang

**Affiliations:** 1 Department of Geriatric Respiratory and Sleep, The First Affiliated Hospital of Zhengzhou University, No.1, Jianshe East Road, Erqi District, Zhengzhou, 450000 Henan Province China; 2 Department of Anesthesiology, The First Affiliated Hospital of Zhengzhou University, Zhengzhou, China

**Keywords:** Baicalin, Chronic obstructive pulmonary disease, Inflammation, Heat shock protein 72, c-Jun N-terminal kinase signaling

## Abstract

**Background:**

Chronic obstructive pulmonary disease (COPD) is characterized by airway obstruction and progressive lung inflammation. As the primary ingredient of a traditional Chinese medical herb, Baicalin has been previously shown to possess anti-inflammatory abilities. Thus, the current study aimed to elucidate the mechanism by which baicalin alleviates COPD.

**Methods:**

Baicalin was adopted to treat cigarette smoke in extract-exposed MLE-12 cells after which cell viability and apoptosis were determined. The production of tumor necrosis factor alpha (TNF-α), interleukin-6 (IL-6), IL-8 were determined by enzyme-linked immunoassay. A COPD mouse model was constructed via exposure to cigarette smoke and lipopolysaccharide, baicalin treatment. Lung function and inflammatory cell infiltration were determined and the production of Muc5AC, TNF-α, IL-6, IL-8 in the bronchoalveolar lavage fluid (BALF) was assayed by ELISA. The effect of HSP72 and JNK on COPD following treatment with baicalin was assessed both *in vivo* and *in vitro* by conducting loss- and gain- function experiments.

**Results:**

Baicalin improved lung function evidenced by reduction in inflammatory cell infiltration and Muc5AC, TNF-α, IL-6 and IL-8 levels observed in BALF in mice. Baicalin was further observed to elevate cell viability while inhibited apoptosis and TNF-α, IL-6 and IL-8 levels in MLE-12 cells. Baicalin treatment increased HSP72 expression, while its depletion reversed the effect of baicalin on COPD. HSP72 inhibited the activation of JNK, while JNK activation was found to inhibit the effect of baicalin on COPD.

**Conclusions:**

Baicalin upregulated the expression of HSP72, resulting in the inhibition of JNK signaling activation, which ultimately alleviates COPD.

**Supplementary Information:**

The online version contains supplementary material available at 10.1186/s10020-021-00309-z.

## Background

Chronic obstructive pulmonary disease (COPD) remains a troubling public health issue worldwide, characterized by airway obstruction and progressive lung inflammation secondary to the influx of proinflammatory cells (Labaki and Rosenberg 2020), accompanied by an increased production of proinflammatory factors including various inflammatory interleukins (ILs), tumor necrosis factor alpha (TNF-α) and matrix metallopeptidase 9 (MMP9) (Barnes 2016). COPD poses a significant threat to global public health owing to its high prevalence and mortality on a global scale (Disease et al. 2016). Cigarette smoke (CS) has been widely documented as the chief risk factor involved in the development of COPD (Scanlon et al. 2000). Studies have indicated that CS contains 10^14^-10^16^ free radicals, including reactive oxygen species and reactive nitrogen species per puff, which ultimately inflicts cellular damage within the lungs secondary to the disruption of the oxidant-antioxidant balance (Pryor et al. 1983). Herbal-based therapies have become increasingly popular therapeutic approaches due to their effectiveness and favorable side effect profiles (Lee et al. 2014). Baicalin is the main effective ingredient found in a traditional Chinese medical herb, *Scutellaria baicalensis Georgi*. Previous studies have highlighted the function of Baicalin as an anti-inflammatory (Yang et al. 2013), antioxidant (Zhou et al. 2012), and antibacterial (Wu et al. 2008) molecule. Due to its potential ability to improve lung function, Baicalin has been speculated to be a potential treatment option for COPD (Wang et al. 2018).

Interestingly, baicalin was also reported to upregulate the expression of heat shock protein 72 (HSP72) and decreased inflammatory response (Yang et al. 2016). As a member of the HSP family, HSP72 is a molecular chaperone (Mayer and Bukau 2005) that has been shown to play a crucial role in the process of intracellular inflammation and apoptotic signaling pathway (Yenari et al. 2005). HSP72 exerts its anti-apoptotic effects via inhibition of caspases as well as inhibition of the extrinsic apoptosis pathway (Powers et al. 2009). Overexpression of HSP72 reportedly inhibits the production of lipopolysaccharide (LPS)-induced inflammatory cytokines resulting in a decrease in inflammatory injury in murine immune cells (Muralidharan et al. 2014). Moreover, COPD patients have been observed exhibit both upregulated activity and phosphorylation of c-Jun N-terminal kinase (JNK) (Eurlings et al. 2017); knockdown of HSP72 has been reported to elevate the activity and phosphorylation of JNK (Kitano et al. 2019), which may represent a potential mechanism by which HSP72 protects against inflammatory injury in the lung. However, the exact mechanism by which baicalin exerts its protective role against COPD is yet to be elucidated. Hence, the current study aimed to investigate the role of baicalin on inflammation in COPD mouse model via evaluation of the effects exerted by baicalin on the expression of HSP72 and JNK signaling pathway in an attempt to ascertain the potential associated mechanisms.

## Materials and methods

### Baicalin

Baicalin (Synonym: 5, 6-Dihydroxy-7-*O*-β-D-glucopyranosyl-2-penyl-4 H-1-benzopyran-4-one, C_21_H_18_O_11_, CAS: 21967-41-9) was purchased from Solarbio (Beijing, China). The purity of the baicalin was confirmed to be greater than 98% based on the findings of a high performance liquid chromatograph.

### Mouse models of the CS exposure and LPS-induced airway inflammation

Model establishment was performed based on a previously reported method (Shin et al. 2017). A total of 80 C57BL/6 specific-pathogen-free male mice (SPF; 6–8 weeks old and 18–25 g) were obtained from SJA Laboratory Animal Co., Ltd. (Hunan, China). Mice were randomly divided into ten groups (8 mice/group), and housed in a SPF grade animal facility with free access to water and food. CS was produced using 3R4F research cigarette (Kentucky reference cigarette; Center for Tobacco Reference Products, University of Kentucky, USA). The mice were exposed to CS in an exposure chamber for 1 h each day over a period of 7 days (Shin et al. 2017). LPS (10 µg/mouse) (Sigma, St. Louis, MO, USA) was administered to animals via nasal instillation on the 4th day of anesthesia. Baicalin, a flavonoid extracted and isolated from the roots of Scutellaria baicalensis, has been reported to exert significant biological activity and provide protection against respiratory inflammation elicited by CS both *in vivo* and *in vitro*, which is generally treated by gavage (Li et al. 2012; Wang et al. 2018; Lixuan et al. 2010). Thus, baicalin was delivered into the animals at doses of 25 mg/kg, 50 mg/kg or 100 mg/kg by intragastric administration in 0.3 mL volume 1 h for 7 d before CS exposure (Li et al. 2012). Oral administration of CCT251236 (MCE, HY-101,026, 20 mg/kg) or intraperitoneal injection of Anisomycin (MCE, HY-18,982, 0.1 mg/kg) was also performed.

### Evaluation of lung function

The mice were anesthetized by means intraperitoneal injection of 2% pentobarbital (40 µg/g). The mice were subsequently placed in a computer-connected forced pulmonary manoeuvre system (Buxco, NY, USA) with an average breathing frequency of 150 breaths/min enforced on the mice. The functional residual capacity (FRC) was detected by compelling the mice to breath against a closed valve. A standard pressure of + 30 cm H_2_O was inflated to the lungs to measure the total lung capacity (TLC), with the pressure slowly decreased to that of -30 cm H_2_O. The forced vital capacity (FVC) and forced expiratory volume within 1 s were measured simultaneously.

### Pulmonary histopathology

The left lung of the mice was collected and washed with saline in order to clean the blood surrounding the lung. The lungs were fixed in 4% paraformaldehyde (Leagene, Beijing, China) for 24 h, embedded in paraffin and stained with hematoxylin and eosin (H&E) (Beyotime, Shanghai, China). Histopathological assessment of the bronchus and the parenchyma was performed in a blind manner. The severity of inflammation was scored on a scale of 0–3 according to the following: 0 = no inflammatory response; 1 = mild inflammation with foci of inflammatory cells in bronchial or vascular wall and in alveolar septa; 2 = moderate inflammation with patchy inflammation or localized inflammation in the walls of the bronchi or blood vessels and alveolar septa, with involvement of less than one-third of the total cross-sectional area of the lung; 3 = severe inflammation with evidence of diffuse inflammatory cell infiltration in the walls of the bronchi or blood vessels and alveoli septa with involvement of one-third to two-third of the lung area.

The change of air space size was assessed using a previously reported method of determining the mean linear intercepts (MLI) (Sato et al. 2006). The number of alveolar septum was calculated in ten randomly selected fields in each section. The destructive index (DI) was calculated to evaluate the destruction of the alveolar wall (Saetta et al. 1985). Ten randomly selected fields in each section were evaluated in order to determine the DI.

### **Immunohistochemistry**

Paraffin-embedded lobe tissue sections were collected from the left lung, de-waxed, dehydrated using alcohol of gradient concentrations. The sections were subsequently immersed in 3% H_2_O_2_ for 20 min and rinsed using distilled water for 2 min and 0.1 M phosphate buffer saline (PBS) for an additional 3 min followed by antigen retrieval in a water bath. The tissue sections were then blocked with normal goat serum (C-0005, Shanghai Haoran Biotech Co., Ltd., Shanghai, China) at room temperature for 20 min, probed with primary antibody to HSP72 (ab2787, 1:100, Mouse, Abcam Inc., Cambridge, MA, USA), phosphorylated (p)-JNK (4668, 1:50, Rabbit, CST, Danvers, MA, USA) at 4 ℃ overnight and re-probed with secondary antibody of goat anti-rabbit or goat anti-mouse immunoglobulin G (IgG) (Boster Co., Ltd., Wuhan, Hubei, China) at 37 ℃ for 20 min. The sections were then incubated with horseradish peroxidase (HRP)-conjugated streptavidin-ovalbumin working solution (0343-10000U, Imunbio Co., Ltd., Beijing, China) at 37℃ for 20 min, developed using 3,3′-diaminobenzidine (ST033, Whiga Co., Ltd., Guangzhou, Guangdong, China), counterstained with hematoxylin (PT001, Bogoo Biotech Co., Ltd., Shanghai, China) for 1 min, immersed in 1% ammonia to facilitate color change to blue, dehydrated by alcohol, cleared by xylene, and mounted with neutral resin, followed by microscopic observation.

### Enzyme-linked immunoassay (ELISA)

After anesthesia, a tracheostomy was performed in the mice as previously based on a previously reported method (Shin et al. 2017). Briefly, bronchoalveolar lavage fluid (BALF) was collected and the cells were resuspended in PBS. The suspended cells were spread onto glass microscope slides, air-dried and then stained with Diff-Quik® reagen (IMEB Inc, San Marcos, CA, USA). The amount of Muc5AC, TNF-α, IL-8 and IL-6 in the BALF were subsequently determined using ELISA kits (R & D System, CA, USA) based on the manufacturer’s instructions. Absorbance values were measured at 450 nm using a microplate reader (BioRad, CA, USA).

### Preparation of aqueous cigarette smoke extract (CSE)

In order to reproduce the effect of cigarette smoke on cells, aqueous CSE was prepared by inflammation of an unfiltered cigarette on a peristaltic pump. The smoke was bubbled into 10 mL of medium within 5 min, followed by pH adjusting to 7.4. The medium was subsequently filtered using a 0.22 μm syringe filter for the removal of bacteria and large particles. The concentration of CSE in the medium was defined as 10% and the prepared CSE was used within 30 min.

### Cell treatment and grouping

Mouse lung type II epithelial cells (MLE-12, American Type Culture Collection, Manassas, VA) were cultured in HITES medium (50:50; Dulbecco Modified Eagle Medium: Ham’s F-12; Gibco, Carlsbad, CA, USA) supplemented with 2% fetal bovine serum (Gibco), 2 mM L-glutamine (Thermo Fisher, Austin, Texas, USA), 10 mM 4-(2-hydroxyerhyl)piperazine-1-erhanesulfonic acid (Thermo), 1:100 insulin/transferrin/selenium supplement (Invitrogen, Carlsbad, CA, USA) and antibiotics/antimycotics (Gibco).

The cells were cultured in HITES medium containing at varying concentrations (5, 10, 20 µmol/L) of baicalin for 24 h, followed by stimulation with CSE (5%) for 2 h.

The small interfering RNAs (siRNAs) (si-negative control [NC], and si-HSP72) were procured from from Ruibio Biotech Co., Ltd., (Beijing, China), with the overexpression vectors (oe-NC, and oe-HSP72) constructed by TsingKe Biotech Co., Ltd., (Beijing, China). The vectors were transfected into MLE-12 cells using lipofectamine 3000 (Invitrogen, USA) when the cells reached 70% confluency. In addition, Anisomycin (4 µM, JNK activator) and SP600125 (JNK inhifbitor, 10 µM, ab120065, Abcam) were introduced for cell treatment (Chen et al. 2020).

**Fig. 1:**
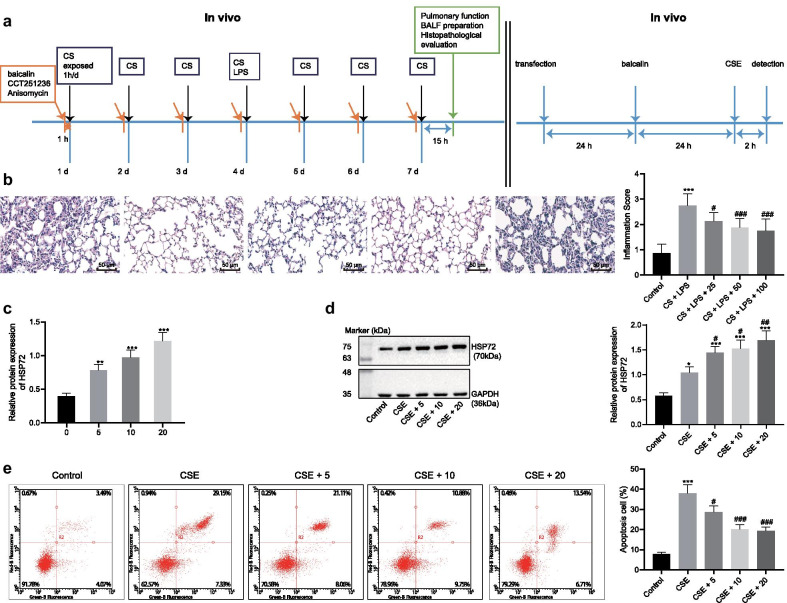
Baicalin alleviates COPD and upregulates the expression of HSP72. COPD was induced by exposing the mice to CS and LPS. **a** Flow-process diagrams of in vivo and in vitro experiments. **b** Inflammatory cell infiltration in the lung was analyzed by H&E staining (×200) and the severity of inflammation was scored. **c** The expression of HSP72 in MLE-12 cells after baicalin treatment for 24 h, ^*^p < 0.05 compared to mice treated with 0 µmol baicalin. **d** The expression of HSP72 normalized to GAPDH was analyzed using immunoblotting after MLE-12 cells were treated with baicalin for 24 h and stimulated by CSE for 2 h. **e** Cell apoptosis of MLE-12 cells were determined using flow cytometry. ^*^p < 0.05, ^**^p < 0.01, ^***^p < 0.0001 compared to normal mice, ^#^*p* < 0.05, ^##^*p* < 0.01, ^###^*p* < 0.001 compared to mice treated with CSE. All experiments were repeated three times independently and the data were represented as mean ± standard deviation. One-way ANOVA followed by Tukey’s test was used for multiple group comparison

Cells in vitro were assigned into the following groups of experiments: (1) control, CSE, CSE + si-NC, CSE + si-HSP72; (2) control, CSE, CSE + baicalin,CSE + baicalin + si-HSP72; (3) control, CSE, CSE + SP600125, CSE + SP600125 + si-HSP72; (4) CSE, CSE + Anisomycin, CSE + baicalin, CSE + baicalin + Anisomycin, CSE + baicalin + Anisomycin + oe-HSP72, and their corresponding NCs. In vitro experiments conducted are depicted in Fig. 1a.

For in vivo study, mice were classified into the following groups of experiments: (1) CS + LPS + baicalin (25 mg/kg), CS + LPS + baicalin (50 mg/kg), CS + LPS + baicalin (100 mg/kg); (2) control, CS + LPS, CS + LPS + baicalin, CS + LPS + baicalin + CCT251236; (3) CS + LPS, CS + LPS + Anisomycin, CS + LPS + baicalin, CS + LPS + baicalin + Anisomycin. The in vivo experiments conducted are illustrated in Fig. 1a.

### Cell counting kit-8 (CCK-8) assay

MLE-12 cells were seeded into a 96-well plate with 5000 cells per well. Cell viability was assessed via CCK-8 (Dojinbo Molecular Technologies, Japan). Briefly, after undergoing cell stimulation and incubation for 1 h at 37℃ in humidified 95% air and 5% CO_2_, the CCK-8 solution was added to the culture medium. Absorbance values were measured at 450 nm using a microplate reader (Bio-Rad, Hercules, CA, USA).

### Flow cytometry

Cell apoptosis analysis was performed using propidium (PI) and fluorescein isothiocyanate (FITC)-conjugated Annexin V staining. Briefly, cells were fixed with 70% ethanol and stained with PI/FITC-Annexin V in the presence of 50 mg/mL RNase A (Sigma), after which incubation for 1 h at room temperature under dark conditions. Flow cytometry was performed using a FAC scan (Beckman Coulter, Brea, CA, USA). The data obtained was analyzed using FlowJo software (Tree Star, USA).

### Reverse transcription quantitative polymerase chain reaction (RT-qPCR)

The total RNA of the cells was extracted by Trizol Reagent (Invitrogen) while complementary DNA (cDNA) was synthesized using Revert Aid first strand cDNA synthesis kit (Fermentas, Life Sciences, Canada) from 1 µg of total RNA. RT-qPCR was performed on an ABI PRISM® 7900HT system (Takara, Japan) using SYBR Premix EXTaq TMII reagent. Glyceraldehyde-3-phosphate dehydrogenase (GAPDH) was employed as the endogenous reference, with the relative expression level of genes determined using the 2^− ΔΔCt^ method. The primers used in this experiment are illustrated in Additional file 1: Table S1.

### Immunoblotting

Protein samples were separated by sodium dodecyl sulfate-polyacrylamide gel electrophoresis and transferred to polyvinylidene fluoride membranes (Immobilon P, Millipore, Billerica, USA). The membrane was blocked with 5% skimmed milk in Tris-buffered saline supplemented with 0.1% Tween-20 for 1 h at room temperature. The primary antibodies were then subject to incubation with the membrane overnight at 4 ℃, followed by three washes of the membrane using Tris-buffered saline Tween (TBST). The HRP-conjugated secondary antibodies were then incubated with the membrane for 1 h at 37 ℃. The antibodies used in this study included mouse anti-HSP72 (1:1000, ab2787, Abcam), rabbit anti-JNK (1:1000, 9252, CST), rabbit anti-p-JNK (1:1000, 4668, CST); anti-rabbit IgG HRP (1:10,000, AS014, Abclonal, Wuhan, Hubei, China) and anti-mouse IgG HRP (1:10,000, AS003, Abclonal). The membrane was subsequently developed with enhanced chemiluminescence reagent (Thermo Fisher) and photographed with ChemiDoc XRS Plus luminescent image analyzer (Bio-Rad). The band intensity was quantified by Image-Pro Plus 6.0 software and then normalized to GAPDH.

### Statistical analysis

All data were analyzed using SPSS 21.0 software (SPSS, Inc., Chicago, IL, USA). Data were expressed as the mean ± standard deviation (mean ± SD) based on three independent tests. Unpaired student’s *t*-test was applied to analyze data displaying normal distribution and equal variance between two groups, while a one-way ANOVA followed by Turkey’s multiple comparisons test were used for analyses between multiple groups. *p* < 0.05 was considered to be indicative of statistical significance.

## Results

### Baicalin increases the expression of HSP72 in lung tissues

We initially established airway inflammation mouse models by exposing the mice to CS and LPS (Fig. 1a), with the mice subsequently treated with baicalin. After the lung function index FEV 0.1/FRC (%), FRC (mL), VC (mL) and TLC (mL) had been determined, the results revealed that CS and LPS triggered obstructive pulmonary ventilation dysfunction in the mice (Additional file 2: Fig. S1A). As expected, the H&E staining results (Fig. 1b) revealed evidence of inflammatory cell infiltration in the airway and lung parenchyma baicalin in the mice following CS and LPS induction, while baicalin treatment was found to alleviate the dysfunction induced by CS and LPS in mice. More specifically, baicalin treatment (50 mg/kg and 100 mg/kg) brought about a distinct reduction in the inflammatory cell infiltration induced by CS and LPS. Additionally, baicalin (50 mg/kg and 100 mg/kg) proved to also be effective in diminishing MLI and DI in CS- and LPS-exposed mice (Additional file 2: Fig. S1B, C). These results highlighted the successful establishment of the airway inflammation mouse model and that baicalin treatment could effectively alleviate the manifestations of COPD.

We subsequently set out to determine the expression of HSP72 in mouse lung tissues with the application of immunoblotting. The expression of HSP72 elevated in CS- and LPS-exposed mice compared to control mice (Additional file 2: Fig. S1D). Besides, the expression of HSP72 was also markedly elevated in baicalin-treated mice (Additional file 2: Fig. S1D). Next, to confirm the effect of baicalin, an in vitro cell model was induced by means of stimulating MLE-12 cells using CSE at different concentrations over a period of 2 h, with the appropriate concentration of CSE selected for subsequent experiments. Our data revealed that CSE (5%) resulted in approximately 50% cell survival rate (Additional file 2: Fig. S1E). The MLE-12 cells were treated with different doses of baicalin (5, 10, 20 µmol/L) for 24 h, with upregulated HSP72 expression observed followed by baicalin treatment (Fig. 1c). We subsequently treated the MLE-12 cells with baicalin for 24 h and CSE stimulation for 2 h. The results obtained indicated that both CSE and baicalin elevated the expression of HSP72 relative to that of the cells without any treatment (Fig. 1d) while CSE was found to induce cell apoptosis whereas baicalin inhibited apoptosis (Fig. 1e). Taken together, these results indicated that baicalin could alleviate COPD by means of upregulating HSP72.

### Knockdown of HSP72 exacerbates CS-induced injury on MLE-12 cells

**Fig. 2:**
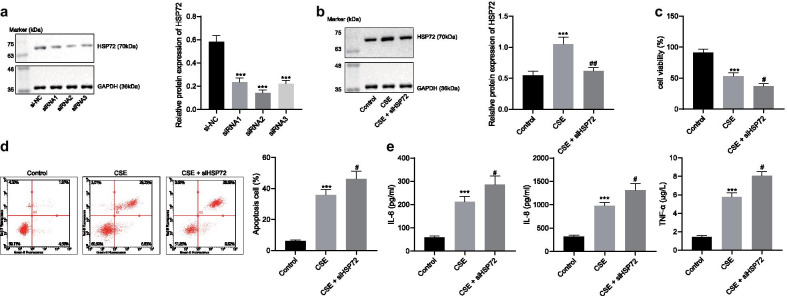
Knockdown of HSP72 exacerbates CS-induced injury on MLE-12 cells. **a** Relative protein expression of HSP72 following treatment of HSP72 siRNA normalized to GAPDH was evaluated by immunoblotting; *p < 0.05, **p < 0.01, ***p < 0.001 compared to cells transfected with si-NC. **b** MLE-12 cells was transfected with si-HSP72 and stimulated with 5% CSE, and the expression of HSP72 normalized to GAPDH was detected by immunoblotting. CSE was exposed to control and HSP72 knocked down cells. **c** Cell viability was tested with CCK-8 assay. **d** Cell apoptosis was tested with flow cytometry. **e** The release of IL-6, IL-8, and TNF-α was detected by ELISA, *p < 0.05, **p < 0.01, ***p < 0.001 compared to untreated cells, ^#^*p* < 0.05, ^##^*p* < 0.01, ^###^*p* < 0.001 compared to cells treated with CSE. All experiments were repeated for three times independently and the data were represented as mean ± standard deviation. One-way ANOVA followed by Tukey’s test was used for multiple group comparison

Next, to further elucidate the role of HSP72 in COPD, we knocked down HSP72 in MLE-12 cells via siRNA. Immunoblotting illustrated that all three si-HSP72 sequences markedly decreased the expression of HSP72 with si-HSP72-2 providing indication of the most efficient one (Fig. 2a). Thus, si-HSP72-2 was used in subsequent studies. CSE (5%) markedly upregulated the expression of HSP72 in MLE-12 cells relative to that of the untreated cells, while the expression of HSP72 was downregulated in cells upon HSP72 knockdown (Fig. 2b). CSE stimulation also decreased cell viability and increased apoptosis compared to unstimulated cells, and knockdown of HSP72 resulted in further inhibition of cell viability and enhanced apoptosis of CSE-stimulated cells (Fig. 2c, d). ELISA was then used to determine the levels of IL-6, IL-8 and TNF-α in the culture medium. The results showed that the levels of IL-6, IL-8 and TNF-α were upregulated in CSE-stimulated cells compared to untreated cells (Fig. 2e). On the other hand, knockdown of HSP72 further increased the expression of IL-6, IL-8 and TNF-α in CSE-challenged MLE-12 cells. Thus, HSP72 knockdown enhanced apoptosis and inflammatory factor release induced by CSE.

### Baicalin alleviates COPD in an HSP72 dependent manner

**Fig. 3:**
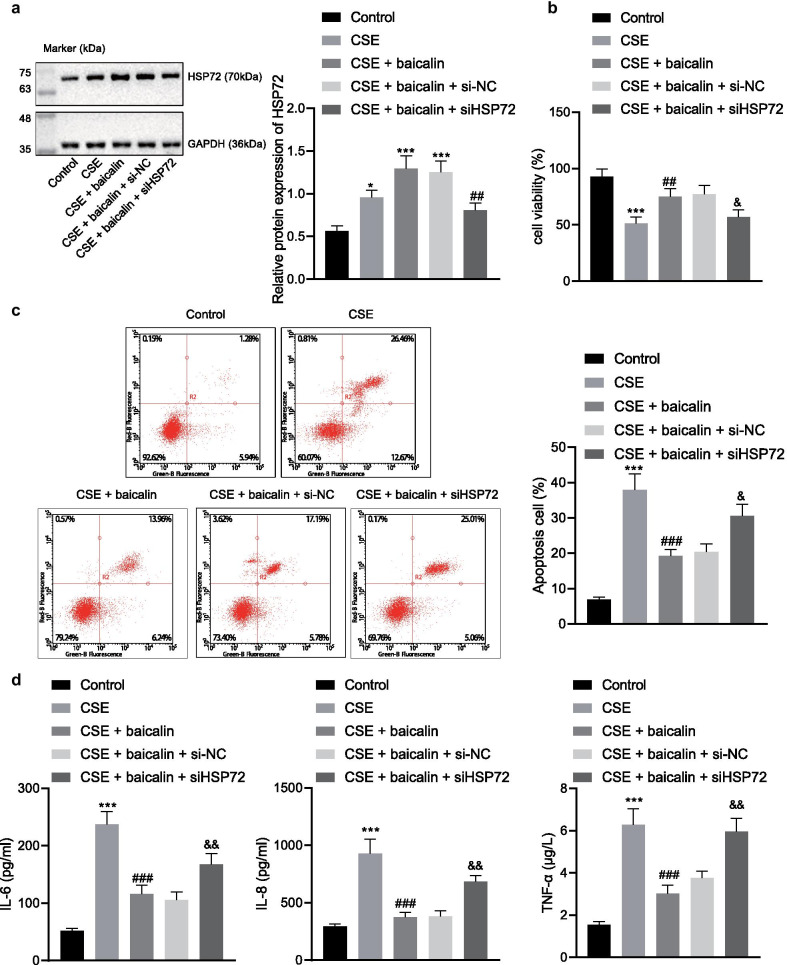
Knockdown of HSP72 retards the effect of baicalin on the treatment of COPD. Cells were treated with CSE, CSE + baicalin, CSE + baicalin + si-NC, or CSE + baicalin + si-HSP72. Mice were treated with CS + LPS, CS + LPS + baicalin, or CS + LPS + baicalin + CCT251236. **a** The expression of HSP72 normalized to GAPDH was detected by immunoblotting in cells; *p < 0.05, **p < 0.01, ***p < 0.001 compared to untreated cells; ^#^*p* < 0.05, ^##^*p* < 0.01, ^###^*p* < 0.001 compared to cells treated with CSE + baicalin + si-NC. **b** The viability of the above cells was determined by CCK-8; *p < 0.05, **p < 0.01, ***p < 0.001 compared to untreated cells; ^#^*p* < 0.05, ^##^*p* < 0.01, ^###^*p* < 0.001 compared to cells treated with CSE; ^&^*p* < 0.05, ^&&^*p* < 0.01, ^&&&^ *p* < 0.001 compared to cells treated with CSE + baicalin + si-NC. **c** Flow cytometry was used to analyze apoptosis of the cells. **d** Release of inflammatory factors IL-6, IL-8 and TNF-α was measured by ELISA

To study the underlying mechanism of baicalin on the development of COPD, we treated CSE-exposed MLE-12 cells with baicalin and detected the expression of HSP72 with the use of immunoblotting. The results revealed that the expression of HSP72 was markedly upregulated by baicalin and CSE-treated cells compared to untreated cells (Fig. 3a). CCK-8 and flow cytometry results indicated that CSE decreased cell viability while enhancing cell apoptosis and this effect was reversed by baicalin treatment in MLE-12 cells. Silencing of HSP72 inhibited the promotion effect of baicalin on cell viability, while inhibiting the effect of baicalin on cell apoptosis (Fig. 3b, c). Moreover, baicalin was further observed to decrease the expression of IL-6, IL-8, and TNF-α upregulated by CSE in untreated cells, but HSP72 knockdown reversed the inhibitory role of baicalin on IL-6, IL-8 and TNF-α in cells (Fig. 3d).

**Fig. 4:**
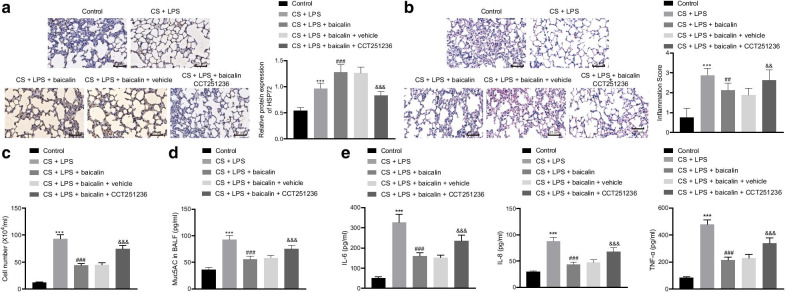
The HSP72 inhibitor CCT251236 inhibited the therapeutic effect of baicalin on COPD in vivo. **a** HSP72 expression in the lung tissues of mice was tested by immunohistochemistry (×400). **b** Lung injury was visualized by H&E staining (×400) and lung injury was scored. **c** The white blood cells in the BALF were measured by ELISA. **d** Muc5AC expression in the BALF was determined. **e** Expression of IL-6, IL-8, TNF-α in the BALF was measured by ELISA. Compared with the control group, *p < 0.05, **p < 0.01, ***p < 0.001; compared with the CS + LPS group, ^#^*p* < 0.05, ^##^*p* < 0.01, ^###^*p* < 0.001; compared with the CS + LPS + baicalin + vehicle group, ^&^*p* < 0.05, ^&&^*p* < 0.01, ^&&&^ *p* < 0.001. All experiments were repeated for three times independently and the data were represented as mean ± standard deviation. One-way ANOVA followed by Tukey’s test was used for multiple group comparison

Next, to ascertain the effect of baicalin on COPD is dependent on HSP72 in vivo, we administrated HSP72 inhibitor CCT251236 (20 mg/kg) to inhibit the expression of HSP72 in mice and tested the effect of baicalin on COPD. CS and LPS which led to an upregulation of HSP72 in lung tissues of mice and baicalin treatment further increased HSP72 expression yet oral administration of CCT251236 significantly inhibited HSP72 expression in mouse lung tissues according to immunohistochemistry results (Fig. 4a). Furthermore, our data revealed that CS and LPS induced lung dysfunction while baicalin improved this dysfunction in the normal mice. Moreover, in the CCT251236-administrated mice, the efficacy of baicalin was markedly reduced which was accompanied by aggravated lung dysfunction (Additional file 3: Fig. S2A). H&E staining illustrated inflammatory cell infiltration in the lung after lung injury, whereas inflammatory cell infiltration was reduced by baicalin (50 mg/kg) treatment in normal mice. Compared to vehicle control-administrated mice, the CCT251236 administration increased inflammatory cell infiltration in lung in mice treated with CS + LPS + baicalin (Fig. 4b). In addition, baicalin (50 mg/kg) was found to effectively diminish the MLI and DI of CS and LPS-exposed mice, but the effect was inhibited by CCT251236 treatment (Additional file 3: Fig. S2B, C). The amount of white blood cells as well as Muc5AC, IL-6, IL-8, and TNF-α in the BALF all exhibited increases post lung injury. Baicalin effectively lowered the amount of the cells and inflammatory factors in the BALF of CS and LPS-exposed mice, but the effect was reversed by CCT251235 treatment (Fig. 4c-e). Thus, we concluded that baicalin could alleviate COPD in a HSP72 dependent manner.

### HSP72 inhibits the JNK pathway which exacerbates COPD

**Fig. 5:**
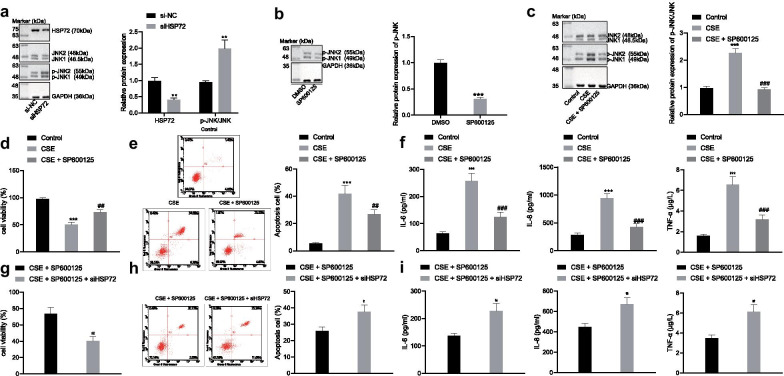
HSP72 inhibits COPD through the inhibition of the JNK signaling pathway. **a** MLE-12 cells were transfected with si-HSP72 and the expression of HSP72 and JNK phosphorylation normalized to GAPDH was analyzed by immunoblotting; *p < 0.05, **p < 0.01, ***p < 0.001 compared to cells transfected with si-NC. **b** The extent of JNK phosphorylation normalized to GAPDH after SP600125 treatment was analyzed by immunoblotting; *p < 0.05, **p < 0.01, ***p < 0.001 compared to cells treated with DMSO. MLE-12 cells were treated with SP600125 and stimulated with CSE. **c** The phosphorylation of JNK normalized to GAPDH was analyzed by immunoblotting. **d** Cell viability was detected by CCK-8 assay. **e** Cell apoptosis was detected by flow cytometry. **f** Release of IL-6, IL-8, and TNF-α was analyzed by ELISA. ^*^p < 0.05, ^**^p < 0.01, ^***^p < 0.001 compared to untreated cells; ^#^*p* < 0.05, ^##^*p* < 0.01, ^###^*p* < 0.001 compared to cells treated with CSE. MLE-12 cells were co-treated with si-HSP72 and SP600125. **g** Cell viability was detected by CCK-8 assay. **h** Cell apoptosis was detected by flow cytometry. **i** Release of IL-6, IL-8, and TNF-α was analyzed by ELISA. *p < 0.05, **p < 0.01,***p < 0.001 compared to CSE + SP600125. All experiments were repeated for three times independently and the data were represented as mean ± standard deviation. One-way ANOVA followed by Tukey’s test was used for multiple group comparison

Subsequently, to elucidate the regulatory role of baicalin on the HSP72-dependent JNK signaling pathway in COPD, we first knocked down HSP72 in MLE-12 cells, the results of which showed that silencing of HSP72 increased phosphorylation of JNK (Fig. 5a). A JNK inhibitor (SP600125) was subsequently applied to downregulate JNK in MLE-12 cells. As per the immunoblotting results, SP600125 led to a significant reduction in the extent of JNK phosphorylation (Fig. 5b). The immunoblotting results revealed that CSE markedly increased JNK phosphorylation in untreated cells, while CSE failed to increase JNK phosphorylation in cells treated with SP600125 (Fig. 5c). CCK-8 and flow cytometry results showed that CSE decreased cell viability and enhanced apoptosis in untreated cells, whereas knock down of JNK recovered the effect of CSE on cell viability and apoptosis (Fig. 5d, e). As depicted in Fig. 5f, ELISA demonstrated that release of IL-6, IL-8 and TNF-α induced by CSE was also decreased following the knockdown of JNK in MLE-12 cells. When si-HSP72 and SP600125 were co-delivered into CSE-treated cells, CCK-8, flow cytometry and ELISA results demonstrated that the cells had decreased cell viability along with a concomitant increase in the rate of apoptosis as well as IL-6, IL-8, and TNF-α expression when compared to the CSE-treated cells treated with SP600125 (Fig. 5g-i). Collectively, these findings indicated that the silencing of JNK reduced apoptosis and inflammatory factors induced by CSE, which was reversed following knockdown of HSP72.

### The inhibition of the JNK pathway of baicalin is dependent on HSP72

**Fig. 6:**
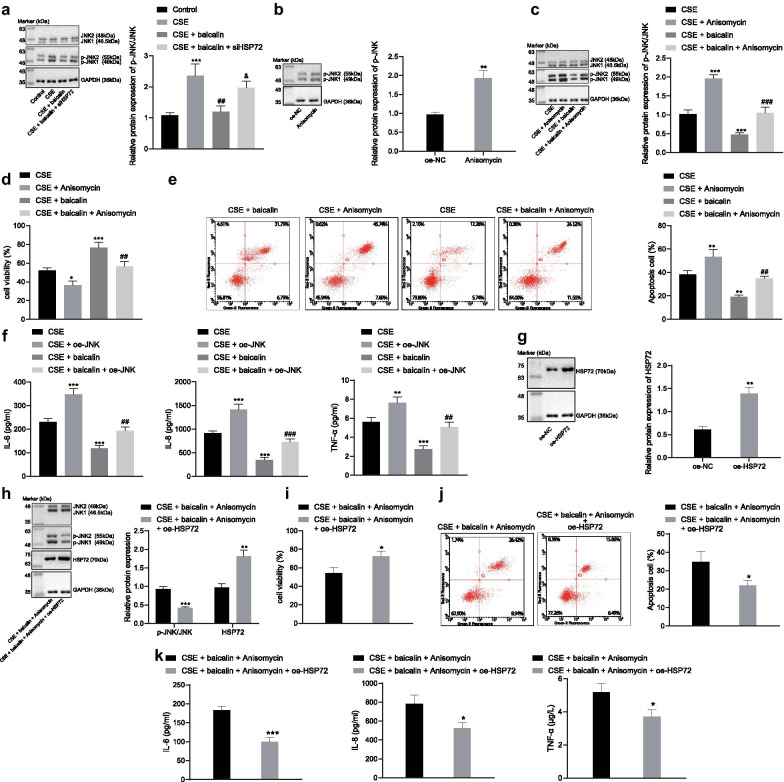
Baicalin blocks the JNK signaling pathway and alleviates COPD in a HSP72 dependent manner. **a** MLE-12 cells were transfected with si-HSP72 or control vector and stimulated with CSE. The cells treated with baicalin and the phosphorylation of JNK normalized to GAPDH was analyzed by immunoblotting; *p < 0.05, **p < 0.01, ***p < 0.001 compared to untreated cells; ^#^*p* < 0.05, ^##^*p* < 0.01, ^###^*p* < 0.001 compared to cells treated with CSE; ^&^*p* < 0.05, ^##^*p* < 0.01, ^###^*p* < 0.001 compared to cells treated with CSE + baicalin. **b** The extent of JNK phosphorylation after Anisomycin treatment normalized to GAPDH was tested by immunoblotting. *p < 0.05, **p < 0.01, ***p < 0.001 compared to cells treated with vehicle. **c** The phosphorylation of JNK normalized to GAPDH was analyzed by immunoblotting. **d** Cell viability was analyzed by CCK-8. **e** Cell apoptosis was analyzed by flow cytometry. **f** Release of IL-6, IL-8 and TNF-α was analyzed ELISA. ^*^p < 0.05, ^**^p < 0.01, ^***^p < 0.001 compared to cells treated with CSE; ^#^*p* < 0.05, ^##^*p* < 0.01, ^###^*p* < 0.001 compared to cells treated with CSE + baicalin. **g** Relative protein expression of HSP72 normalized to GAPDH after treatment of HSP72 overexpression vector was determined by immunoblotting. *p < 0.05, **p < 0.01, ***p < 0.001 compared to cells transfected with oe-NC. **h** Cells were co-transfected with both Anisomycin and oe-HSP72 and stimulated with CSE. The expression of HSP72 and extent of JNK phosphorylation normalized to GAPDH was analyzed by immunoblotting. **i** Cell viability of both JNK-activated and HSP72-overexpressed cells after treatment with baicalin was analyzed by CCK-8 assay. **j** Cell apoptosis of both JNK-activated and HSP72-overexpressed cells after treatment with baicalin was analyzed by flow cytometry. **k** Release of IL-6, IL-8 and TNF-α of both JNK-activated and HSP72-overexpressed cells after treatment with baicalin was analyzed ELISA. *p < 0.05, **p < 0.01, ***p < 0.001 compared to cells treated with CSE + baicalin + Anisomycin. All experiments were repeated for three times independently and the data were represented as mean ± standard deviation. One-way ANOVA followed by Tukey’s test was used for multiple group comparison

Next, to verify whether baicalin could improve COPD by regulating the JNK signaling pathway, we treated MLE-12 cells stimulated by CSE with baicalin and/or si-HSP72 and analyzed the phosphorylation of JNK with the application of immunoblotting. The data obtained indicated that baicalin triggered a marked reduction in the phosphorylation of JNK, while si-HSP72 further decreased the inhibitory effect of baicalin on the phosphorylation of JNK (Fig. 6a). Additionally, the effect of baicalin was evaluated in cells treated with JNK activator (Anisomycin). The extent of JNK phosphorylation was significantly upregulated by Anisomycin, as confirmed by immunoblotting (Fig. 6b). We adopted baicalin and/or Anisomycin to treat CSE-stimulated cell, and immunoblotting results found Anisomycin led to a distinct increase in the phosphorylation of JNK (Fig. 6c). Moreover, activation of JNK also reduced cell viability, which was accompanied by an increase in apoptosis as well as the release of IL-6, IL-8 and TNF-α induced by CSE. Baicalin inhibited apoptosis and the release of inflammatory factors in CSE-stimulated cells. However, when compared to the CSE-stimulated cells treated with baicalin, CSE-stimulated cells treated with Anisomycin exhibited a decrease in cell viability as well as an increase in apoptosis as well as the release of inflammatory factors (Fig. 6d-f). We subsequently obtained data verifying these results in the mouse airway injury model. JNK agonist Anisomycin (0.1 mg/kg, intraperitoneal injection) markedly increased the phosphorylation of JNK in mice (Additional file 4: Fig. S3A). Increased JNK activity aggravated CS and LPS-induced airway injury, lung dysfunction, inflammatory cell infiltration in the lung and the expression of Muc5AC, IL-6, IL-8, and TNF-α (Additional file 4: Fig. S3B-G).

The increase in the activity and phosphorylation of JNK resulted in the inhibition of the baicalin effect on COPD. In order to ascertain whether HSP72 was required for the regulation of JNK and the subsequent COPD by baicalin, we constructed oe-HSP72 vector and its transfection efficiency was verified by immunoblotting (Fig. 6g). The phosphorylation of JNK was found to be decreased in the cells overexpressing HSP72 (Fig. 6h). Moreover, the CSE-stimulated cells treated with baicalin and activated JNK and overexpressed HSP72 displayed increased cell viability (Fig. 6I), decreased apoptosis (Fig. 6j) and IL-6, IL-8 and TNF-α release (Fig. 6k). Taken together, the aforementioned results demonstrated that baicalin increased the expression of HSP72, which led to a blockade in JNK signaling pathway, ultimately alleviating COPD.

## Discussion

**Fig. 7:**
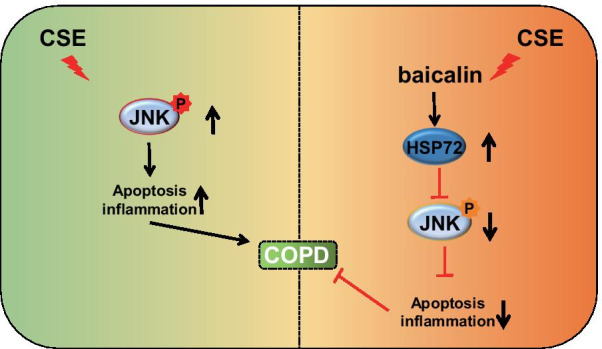
Graphic mechanism. Aqueous CSE stimulation promotes the activation of the JNK pathway thereby promoting the development of COPD, while baicalin can up-regulate the expression of HSP72, which in turn inhibits the activity of the JNK pathway and impedes the development of COPD

COPD has been well documented to be triggered by a set of pathophysiological mechanisms that result in excessive mucus secretion and limited airflow. Smoking has been strongly implicated as the chief risk factor associated with the development of COPD (Hattab et al. 2016). A previous study investigating rat models demonstrated that baicalin could exert inhibitory effects on inflammation, highlighting its protective role against ischemia-reperfusion injury induced by LPS in intestinal epithelial cells and intercellular tight junctions (Chen et al. 2015). During the current study, our results revealed that baicalin upregulated the expression of HSP72, which resulted in the inhibition of the phosphorylation of JNK. This mechanism decreased the production of pro-inflammatory factors and apoptosis, thus serving as a defense method against COPD (Fig. 7).

Initially, CS exposure was observed to trigger a notable increase in inflammatory cell counts, resulting in continuous airway remodeling. Previous literature has indicated that CS exposure inflicts direct damage to airway epithelium in addition to activating macrophages and lymphocytes, generating pro-inflammatory cytokines, thus aggravating COPD (Nakamura et al. 2015). Moreover, the production of the proinflammatory cytokines has been linked with exacerbation of the inflammatory response that occurs during COPD development (Ge et al. 2016). In our subsequent experiment, our findings demonstrated that treatment with baicalin alleviated the degree of inflammation in addition to diminishing the release of TNF-α, IL-6 and IL-8, thus attenuating COPD. A previous study reported that baicalin could diminish levels of pro-inflammatory mediators, TNF-α, IL-6 and IL-1β, in acute lung injury, functioning as a potential therapeutic candidate, which was largely consistent with the observations of our study (Meng et al. 2019; Peng et al. 2019). Baicalin has been previously reported to exhibit no significant toxicity at doses between 20 and 200 mg/kg (Xue et al. 2010), thus three dosages of baicalin (25, 50 and 100 mg/kg) were administrated to treat CS-exposure mice in our study. Consistently, baicalin has been reported to attenuate CS-induced inflammation in COPD rat model via the inhibition of IL-6, IL-8 and TNF-α productions (Lixuan et al. 2010). Inflammatory scores, MLI and DI have all been positively linked with severe lung injury and the improvement of lung function decreases MLI and DI (Jiang et al. 2020). Our in vivo experiments data illustrated that baicalin was capable of diminishing inflammatory scores, MLI, and DI in CS-exposure mice.

Moreover, additional data obtained highlighted the anti-inflammatory effect of baicalin in COPD occurs via the upregulation of HSP72, which further decreased TNF-α, IL-6 and IL-8 levels, and apoptosis, while increasing cell viability. Yang et al. concluded that baicalin could upregulate the expression of HSP72 which led to a decrease in the inflammatory response in cow mammary epithelial cells (Yang et al. 2016). Furthermore, treatment with baicalin, at different concentrations, was found to elevate the expression of HSP72 in primary cultured bovine sertoli cells under exposure to heat stress (Guo et al. 2015). The cytoprotective role of HSP72 has been well documented both as an anti-inflammatory as well as an anti-apoptotic molecule (Giffard et al. 2008). The upregulation of HSP72 has been reported to not only inhibits the NF-κB pathway by stabilizing IκB-NF-κB complex in microglial cell line (Sheppard et al. 2014) but also diminishes the production of proinflammatory cytokines in rat inflammatory models (Dokladny et al. 2010). HSP72 expression has been shown to exhibit increased levels in collagen-induced arthritis mouse model and recombinant HSP72 which markedly inhibits the expression of IL-6, TNF-α and NF-κB, highlighting its protective role in arthritis (Luo et al. 2011).

Furthermore, silencing of HSP72 was shown to increase JNK activity while silencing of JNK decreased TNF-α, IL-6 and IL-8 levels, and apoptosis, while elevating cell viability. Reports have suggested that the knockout of HSP72, a natural inhibitor of JNK leads to an increase in JNK activity (Park et al. 2001; Kitano et al. 2019) while patients with severe COPD have been noted to exhibit upregulated levels of JNK in their adipose tissues (Tkacova et al. 2011). Consistently, patients with COPD displayed increased numbers of positive cells for phosphorylated JNK in epithelial cells (Eurlings et al. 2017). The inhibition or depletion of JNK has been reported to significantly weaken TNF-α-induced lung epithelial cell inflammatory responses (Yu et al. 2010). The overexpression of JNK induces cell migration and invasion, and morphologic changes in human cancer cells (Wang et al. 2010). The expression of HSP72 is directly regulated by baicalin in our study, implicating JNK as a downstream target for baicalin in treatment of COPD.

## Conclusion

The central findings of the current study present evidence documenting the anti-inflammatory effects of baicalin on CS-induced inflammatory models in both mice as well as in MLE-12 cells. Our data lends support to the hypothesis that baicalin plays a crucial role in regulating HSP72, resulting in the attenuation of CS-induced inflammation. The downstream target molecules of baicalin, including HSP72 and JNK, represent a promising novel target for drug development for COPD therapy. However, further studies are required in order to elucidate the role of baicalin in a clinical setting as well as its role in other potential signaling pathways.

## Supplementary Information

**Additional file 1: Table S1.** Sequence of the primers for RT-qPCR

**Additional file 2: Fig S1. **COPD was induced byexposing the mice to CS and LPS. A, Determination ofmouse pulmonary function based on FEV0.1/FVC ratio, FRC, Vital capacity (VC), and TLClevel. B, Calculation of MLI value. C, Calculation of DI value. D, Western blotanalysis of HSP72 expression inmouse lung tissues. *p < 0.05, **p < 0.01, ***p< 0.001 compared to untreated cells. ^#^*p* < 0.05 comparedwith cells treated with CS + LPS. E, CCK-8 was used todetect MLE-12 cell survival after 2 h of stimulation with differentconcentrations of CSE (0%, 2.5%, 5%, 7.5%, and 10%). ^*^p < 0.05, ^**^p < 0.01, ^***^p< 0.001 compared to cells treated with 0% CSE.

**Additional file 3: Fig S2. **COPD was induced byexposing the mice to CS and LPS. The modeled mice were treated with baicalin and/or CCT251236. A, Determination of mouse pulmonary function based on FEV0.1/FVC ratio,FRC, Vital capacity (VC), and TLC level. B, Calculation of MLIvalue. C, Calculation of DI value.*p < 0.05, **p< 0.01, ***p < 0.001 compared to untreated cells. ^#^*p*< 0.05, ^##^*p* < 0.01, ^###^*p*< 0.001 compared to cells treated with CS + LPS. ^&^*p*< 0.05, ^&&^*p* < 0.01, ^&&&^*p* < 0.001 compared to cells treated with CS + LPS + baicalin+ vehicle.

**Additional file 4: Fig S3. **Baicalin alleviates COPD by inhibiting JNKsignaling pathway in a HSP72-dependent manner. A, Immunohistochemistry wasemployed to measure the expression of p-JNK in lung tissues of mice treatedwith JNK activator (400 ×). B, Determination of mouse pulmonary function basedon FEV0.1/FVC ratio, FRC, Vital capacity (VC), and TLC level. C, H&E staining was used to observe inflammatory cells in lungtissues (400 ×). D, Scoring of the severity of inflammation. E, Counting oftotal leukocyte number in BALF. F, ELISA was used to detect the expression ofMuc5AC in BALF. G, ELISA was used to determine the expression of inflammatoryfactors IL-6, IL-8, and TNF-α in BALF.*p < 0.05, **p< 0.01, ***p < 0.001 compared to cells treated with CS+ LPS. ^#^*p* < 0.05, ^##^*p* < 0.01, ^###^*p*< 0.001 compared to cells treated with CS + LPS + baicalin.

## Data Availability

These data and materials of study is available.

## Abbreviations

JNK: C-Jun N-terminal kinase
COPD: Chronic obstructive pulmonary disease
ELISA: Enzyme-linked immunoassay
BALF: Bronchoalveolar lavage fluid
CS: Cigarette smoke
HSP72: Heat shock protein 72
LPS: Lipopolysaccharide
FRC: Functional residual capacity
FVC: Forced vital capacity
MLI: Mean linear intercepts
DI: Destructive index
PBS: Phosphate buffer saline
IgG: Immunoglobulin G
CSE: Cigarette smoke extract
CCK-8: Cell counting kit-8
GAPDH: Glyceraldehyde-3-phosphate dehydrogenase

## References

[CR1] Barnes PJ (2016). Inflammatory mechanisms in patients with chronic obstructive pulmonary disease. J Allergy Clin Immunol.

[CR2] Chen J, Zhang R, Wang J, Yu P, Liu Q, Zeng D (2015). Protective effects of baicalin on LPS-induced injury in intestinal epithelial cells and intercellular tight junctions. Can J Physiol Pharmacol.

[CR3] Chen Y, Liu K, Zhang J, Hai Y, Wang P, Wang H (2020). c-Jun NH2 -Terminal Protein Kinase Phosphorylates the Nrf2-ECH Homology 6 Domain of Nuclear Factor Erythroid 2-Related Factor 2 and Downregulates Cytoprotective Genes in Acetaminophen-Induced Liver Injury in Mice. Hepatology.

[CR4] Disease GBD, Injury I, Prevalence C (2016). Global, regional, and national incidence, prevalence, and years lived with disability for 310 diseases and injuries, 1990–2015: a systematic analysis for the Global Burden of Disease Study 2015. Lancet.

[CR5] Dokladny K, Lobb R, Wharton W, Ma TY, Moseley PL (2010). LPS-induced cytokine levels are repressed by elevated expression of HSP70 in rats: possible role of NF-kappaB. Cell Stress Chaperones.

[CR6] Eurlings IM, Reynaert NL, van de Wetering C, Aesif SW, Mercken EM, de Cabo R (2017). Involvement of c-Jun N-Terminal Kinase in TNF-alpha-Driven Remodeling. Am J Respir Cell Mol Biol.

[CR7] Ge LT, Liu YN, Lin XX, Shen HJ, Jia YL, Dong XW, et al. Inhalation of ambroxol inhibits cigarette smoke-induced acute lung injury in a mouse model by inhibiting the Erk pathway. Int Immunopharmacol. 2016;33(90 – 8. 10.1016/j.intimp.2016.02.004 26881857

[CR8] Giffard RG, Han RQ, Emery JF, Duan M, Pittet JF (2008). Regulation of apoptotic and inflammatory cell signaling in cerebral ischemia: the complex roles of heat shock protein 70. Anesthesiology.

[CR9] Guo X, Chi S, Cong X, Li H, Jiang Z, Cao R (2015). Baicalin protects sertoli cells from heat stress-induced apoptosis via activation of the Fas/FasL pathway and Hsp72 expression. Reprod Toxicol.

[CR10] Hattab Y, Alhassan S, Balaan M, Lega M, Singh AC (2016). Chronic Obstructive Pulmonary Disease Crit Care Nurs Q.

[CR11] Jiang JS, Chou HC, Chen CM. Cathelicidin attenuates hyperoxia-induced lung injury by inhibiting oxidative stress in newborn rats. Free Radic Biol Med. 2020;150(23 – 9. 10.1016/j.freeradbiomed.2020.02.005 32057991

[CR12] Kitano S, Kondo T, Matsuyama R, Ono K, Goto R, Takaki Y (2019). Impact of hepatic HSP72 on insulin signaling. Am J Physiol Endocrinol Metab.

[CR13] Labaki WW, Rosenberg SR (2020). Chronic Obstructive Pulmonary Disease. Ann Intern Med.

[CR14] Lee MY, Seo CS, Kim YB, Shin IS, Shin HK (2014). Non-clinical safety assessment of Hwangryunhaedok-tang: 13-week toxicity in Crl:CD Sprague Dawley rats. Regul Toxicol Pharmacol.

[CR15] Li L, Bao H, Wu J, Duan X, Liu B, Sun J (2012). Baicalin is anti-inflammatory in cigarette smoke-induced inflammatory models in vivo and in vitro: A possible role for HDAC2 activity. Int Immunopharmacol.

[CR16] Lixuan Z, Jingcheng D, Wenqin Y, Jianhua H, Baojun L, Xiaotao F (2010). Baicalin attenuates inflammation by inhibiting NF-kappaB activation in cigarette smoke induced inflammatory models. Pulm Pharmacol Ther.

[CR17] Luo X, Zuo X, Mo X, Zhou Y, Xiao X (2011). Treatment with recombinant Hsp72 suppresses collagen-induced arthritis in mice. Inflammation.

[CR18] Mayer MP, Bukau B (2005). Hsp70 chaperones: cellular functions and molecular mechanism. Cell Mol Life Sci.

[CR19] Meng X, Hu L, Li W (2019). Baicalin ameliorates lipopolysaccharide-induced acute lung injury in mice by suppressing oxidative stress and inflammation via the activation of the Nrf2-mediated HO-1 signaling pathway. Naunyn Schmiedebergs Arch Pharmacol.

[CR20] Muralidharan S, Ambade A, Fulham MA, Deshpande J, Catalano D, Mandrekar P (2014). Moderate alcohol induces stress proteins HSF1 and hsp70 and inhibits proinflammatory cytokines resulting in endotoxin tolerance. J Immunol.

[CR21] Nakamura M, Wada H, Honda K, Nakamoto K, Inui T, Sada M, et al. Clarithromycin ameliorates pulmonary inflammation induced by short term cigarette smoke exposure in mice. Pulm Pharmacol Ther. 2015;35(60 – 6. 10.1016/j.pupt.2015.09.005 26363279

[CR22] Park HS, Lee JS, Huh SH, Seo JS, Choi EJ (2001). Hsp72 functions as a natural inhibitory protein of c-Jun N-terminal kinase. EMBO J.

[CR23] Peng LY, Yuan M, Song K, Yu JL, Li JH, Huang JN, et al. Baicalin alleviated APEC-induced acute lung injury in chicken by inhibiting NF-kappaB pathway activation. Int Immunopharmacol. 2019;72(467 – 72. 10.1016/j.intimp.2019.04.046 31035089

[CR24] Powers MV, Clarke PA, Workman P (2009). Death by chaperone: HSP90, HSP70 or both?. Cell Cycle.

[CR25] Pryor WA, Prier DG, Church DF. Electron-spin resonance study of mainstream and sidestream cigarette smoke: nature of the free radicals in gas-phase smoke and in cigarette tar. Environ Health Perspect. 1983;47(345 – 55. 10.1289/ehp.8347345 PMC1569403 6297881

[CR26] Saetta M, Shiner RJ, Angus GE, Kim WD, Wang NS, King M (1985). Destructive index: a measurement of lung parenchymal destruction in smokers. Am Rev Respir Dis.

[CR27] Sato T, Seyama K, Sato Y, Mori H, Souma S, Akiyoshi T (2006). Senescence marker protein-30 protects mice lungs from oxidative stress, aging, and smoking. Am J Respir Crit Care Med.

[CR28] Scanlon PD, Connett JE, Waller LA, Altose MD, Bailey WC, Buist AS (2000). Smoking cessation and lung function in mild-to-moderate chronic obstructive pulmonary disease. The Lung Health Study. Am J Respir Crit Care Med.

[CR29] Sheppard PW, Sun X, Khammash M, Giffard RG (2014). Overexpression of heat shock protein 72 attenuates NF-kappaB activation using a combination of regulatory mechanisms in microglia. PLoS Comput Biol.

[CR30] Shin NR, Ko JW, Park SH, Cho YK, Oh SR, Ahn KS, et al. Protective effect of HwangRyunHaeDok-Tang water extract against chronic obstructive pulmonary disease induced by cigarette smoke and lipopolysaccharide in a mouse model. J Ethnopharmacol. 2017;200(60 – 5. 10.1016/j.jep.2017.02.027 28216440

[CR31] Tkacova R, Ukropec J, Skyba P, Ukropcova B, Pobeha P, Kurdiova T (2011). Increased adipose tissue expression of proinflammatory CD40, MKK4 and JNK in patients with very severe chronic obstructive pulmonary disease. Respiration.

[CR32] Wang J, Kuiatse I, Lee AV, Pan J, Giuliano A, Cui X (2010). Sustained c-Jun-NH2-kinase activity promotes epithelial-mesenchymal transition, invasion, and survival of breast cancer cells by regulating extracellular signal-regulated kinase activation. Mol Cancer Res.

[CR33] Wang G, Mohammadtursun N, Lv Y, Zhang H, Sun J, Dong J (2018). Baicalin Exerts Anti-Airway Inflammation and Anti-Remodelling Effects in Severe Stage Rat Model of Chronic Obstructive Pulmonary Disease. Evid Based Complement Alternat Med.

[CR34] Wu J, Hu D, Wang KX (2008). [Study of Scutellaria baicalensis and Baicalin against antimicrobial susceptibility of Helicobacter pylori strains in vitro]. Zhong Yao Cai.

[CR35] Xue X, Qu XJ, Yang Y, Sheng XH, Cheng F, Jiang EN (2010). Baicalin attenuates focal cerebral ischemic reperfusion injury through inhibition of nuclear factor kappaB p65 activation. Biochem Biophys Res Commun.

[CR36] Yang X, Yang J, Zou H (2013). Baicalin inhibits IL-17-mediated joint inflammation in murine adjuvant-induced arthritis. Clin Dev Immunol.

[CR37] Yang W, Li H, Cong X, Wang X, Jiang Z, Zhang Q, et al. Baicalin attenuates lipopolysaccharide induced inflammation and apoptosis of cow mammary epithelial cells by regulating NF-kappaB and HSP72. Int Immunopharmacol. 2016;40(139 – 45. 10.1016/j.intimp.2016.08.032 27588914

[CR38] Yenari MA, Liu J, Zheng Z, Vexler ZS, Lee JE, Giffard RG (2005). Antiapoptotic and anti-inflammatory mechanisms of heat-shock protein protection. Ann N Y Acad Sci.

[CR39] Yu Y, Chiba Y, Sakai H, Misawa M (2010). Possible involvements of nuclear factor-kappa B and activator protein-1 in the tumor necrosis factor-alpha-induced upregulation of matrix metalloproteinase-12 in human alveolar epithelial A549 cell line. J Pharmacol Sci.

[CR40] Zhou BR, Yin HB, Xu Y, Wu D, Zhang ZH, Yin ZQ (2012). Baicalin protects human skin fibroblasts from ultraviolet A radiation-induced oxidative damage and apoptosis. Free Radic Res.

