# Supplementary material for: Baicalin alleviates chronic obstructive pulmonary disease through regulation of HSP72-mediated JNK pathway
Source: Mol Med. 2021 May 30;27:53. doi: 10.1186/s10020-021-00309-z (PMC8165801; doi:10.1186/s10020-021-00309-z)
Supplement: Supplementary file 1 — Additional file 1: Table S1. Sequence of the primers for RT-qPCR [file 10020_2021_309_MOESM1_ESM.docx]

**Table S1** Sequence of the primers for RT-qPCR

| Genes | Forward primer (5′-3′) | Reverse primer (5′-3′) |
| --- | --- | --- |
| HSP72 | TGGTGCAGTCCGACATGAAG | GCTGAGAGTCGTTGAAGTAGGC |
| JNK | GTGGAATCAAGCACCTTCACT | TCCTCGCCAGTCCAAAATCAA |
| GAPDH | AGGTCGGTGTGAACGGATTTG | GGGGTCGTTGATGGCAACA |

**Notes:** RT-qPCR, reverse transcription quantitative polymerase chain reaction; GAPDH, Glyceraldehyde-3-phosphate dehydrogenase; HSP72, heat shock protein 72; JNK, c-Jun N-terminal Kinase.
